# Modeling the Dynamic Exclusive Pedestrian Phase Based on Transportation Equity and Cost Analysis

**DOI:** 10.3390/ijerph19138176

**Published:** 2022-07-04

**Authors:** Yining Lu, Tao Wang, Zhuangzhuang Wang, Chaoyang Li, Yi Zhang

**Affiliations:** School of Naval Architecture Ocean and Civil Engineering, Shanghai Jiao Tong University, Shanghai 200240, China; luyining@sjtu.edu.cn (Y.L.); alexsnail@sjtu.edu.cn (Z.W.); cyljjf@sjtu.edu.cn (C.L.); darrenzhy@sjtu.edu.cn (Y.Z.)

**Keywords:** traffic safety, exclusive pedestrian phase, pedestrian–vehicle conflict, transportation equity, cost analysis, pedestrian–vehicle interaction

## Abstract

The exclusive pedestrian phase (*EPP*) has proven to be an effective method of eliminating pedestrian–vehicle conflicts at signalized intersections. The existing *EPP* setting conditions take traffic efficiency and safety as optimization goals, which may contribute to unfair interactions between vehicles and pedestrians. This study develops a multiobjective optimization framework to determine the *EPP* setting criteria, with consideration for the tradeoff between transportation equity and cost. In transportation equity modeling and considering environmental conditions, the transportation equity index is proposed to quantify pedestrian–vehicle equity differences. In cost modeling, traffic safety and efficiency factors are converted into monetary values, and the pedestrian–vehicle interaction is introduced. To validate the proposed optimization framework, a video-based data collection is conducted on wet and dry environment conditions at the selected intersection. The parameters in the proposed model are calibrated based on the results of the video analysis. This study compares the performance of the multiobjective evolutionary algorithm based on decomposition (MOEA) and the nondominated sorting genetic algorithm II (NSGA-II) methods in building the sets of nondominated solutions. The optimization results show that the decrease in transportation equity will lead to an increase in cost. The obtained Pareto front approximations correspond to diverse signal timing patterns and achieve a balance between optimizing either objective to different extents. The sensitivity analysis reveals the application domains for the *EPP* and the traditional two-way control phase (*TWC*) under different vehicular/pedestrian demand, yielding rate, and environment conditions. The *EPP* control is more suitable at intersections with high pedestrian volumes and low yielding rates, especially in wet conditions. The results provide operational guidelines for decision-makers for properly selecting the pedestrian phase pattern at signalized intersections.

## 1. Introduction

Pedestrian traffic comprises a large proportion of the total mixed traffic flow at urban intersections [1]. Providing relatively fair time–space resources for pedestrians is critical to improving the overall intersection performance. However, the increasing trend of vehicle-miles traveling has led to a vehicle-based signal phase design, which can hardly meet the rising demand of pedestrians. At traditional two-phase signalized intersections, pedestrian–vehicle interaction can lead to increased delay and even accidents, resulting in casualties and economic losses [2]. A crucial issue at signalized intersections is to mitigate conflicting movements between vehicles and pedestrians. Researchers and engineers have been seeking solutions to make the best tradeoff between vehicles and pedestrians in terms of efficiency and safety. The exclusive pedestrian phase (*EPP*), as one of the common signal phasing approaches, has been applied to promote pedestrianism in downtown areas in many countries. The *EPP* stops upcoming vehicular traffic from all approaches to allow pedestrians to cross any leg of the intersection (diagonally as well as laterally) without pedestrian–vehicle interaction [3]. Most researchers have concluded that determining the reasonable boundary conditions of *EPP* can significantly enhance traffic safety at signalized intersections [4,5].

In the field of traffic signal optimization and phase setting, the mainstream studies on the setting conditions and quantitative criteria of *EPP* fall into three major categories: efficiency-based [6,7], safety-based [3,8], and cost-based studies [9,10]. Numerous studies tend to take either traffic efficiency or safety as the primary optimization objectives for simplification. To systematically integrate the two aspects, Ma et al. [9] developed a cost-based framework through value weight. However, the differences between pedestrians and vehicles are rarely considered under various environmental conditions, resulting in an unfair allocation of time–space resources. First, traffic behavioral models should be further integrated into the optimization of pedestrian phase patterns. Pedestrian–vehicle interaction mechanisms will affect traffic signal models, including the delay model [11], capacity model [12], and signal timing [13,14], thus influencing the implementation of *EPP*. Second, under various environment conditions, the traffic characteristics and behavior will change significantly, which can affect the saturation flow rate, loss of time, as well as pedestrian–vehicle interaction [3,15]. As a result, the obtained cycle length and green times will vary according to environment conditions. Therefore, from the aspect of transportation equity, it is necessary to minimize the differences under various environment conditions, such that pedestrians and vehicles can cross the intersection with comparatively fair time–space resources of the road.

To solve the above issue, this study established quantitative criteria to determine pedestrian phase patterns between the *EPP* and the traditional two-way control phase (*TWC*). A bi-objective optimization model is developed based on transportation equity and cost analysis. In transportation equity modeling, the primary objective is to minimize the difference of saturation between pedestrians and vehicles while considering the environment influences to ensure fair crossing capacity. In cost modeling, traffic safety and efficiency are transformed into monetary values to select the pedestrian phase pattern in the economic framework. The secondary objective is to minimize the total cost of safety and delay with consideration for the pedestrian–vehicle interactions at the intersection. To validate the proposed optimization framework, a video-based data collection was conducted on wet and dry environment conditions at the selected intersection. A nonlinear programming model was formulated and solved by two evolutionary algorithms. This study mainly makes three contributions:

- A transportation equity index (*TEI*) is proposed to quantify the individual differences under different environment conditions. Additionally, vehicle throughput is converted into passenger throughput to reflect transportation equity between different traffic participants.
- Based on the *TEI*, a bi-objective optimization model is established to find the best tradeoff between transportation equity and cost. The proposed cost model considers the pedestrian–vehicle interaction, which is more consistent with the actual situation.
- Considering the yielding rate and environment conditions, sensitivity analysis is conducted to determine the application domain of *EPP*. The results provide operational guidelines for decision-makers to better select the pedestrian phase pattern at signalized intersections.

The rest of this paper is organized as follows: the second section gives an introduction to the *EPP* setting methods, along with research on transportation equity and pedestrian–vehicle interaction; in the third section, the optimization model is proposed; the fourth section provides the data resource and the model solution; the fifth section is numerical and sensitivity analyses; conclusions are made in the last section.

## 2. Literature Review

### 2.1. Pedestrian–Vehicle Interaction Research

The *EPP* and *TWC* are two common pedestrian phase patterns at urban signalized intersections. Many studies have analyzed the setting conditions and quantitative criteria of *EPP* in terms of efficiency and safety [9,10]. Ivan et al. [5] found that the *EPP* was more safety-beneficial than the *TWC* in small towns, but was not as effective in large towns with low pedestrian compliance rates. Wang et al. [7] studied the setting criteria for the *EPP* at two-phase signalized intersections and proposed a new delay estimation model for traffic participants. The setting of the *EPP* had a better effect in the case of constant vehicle volume and a large pedestrian volume [6,7,16]. Many studies [3,7,17] have focused on the *EPP* application condition, but most studies have not taken the pedestrian–vehicle behavior into account to ensure fair interactions, especially in different environment conditions.

Various mechanisms of pedestrian–vehicle interaction conditions will influence the performance of the *EPP*. Studies have been conducted mainly on pedestrian factors [11,18,19,20], vehicle factors [15,21,22], and traffic environment factors [23,24,25]. The first two factors have been widely studied and integrated into traffic models. Most traffic models (such as delay estimation) were based on clear weather and dry pavement, while failing to consider poor weather conditions in metropolitan areas [24,25]. Other research shows that environmental conditions (infrastructure and facilities, weather, temperature, etc.) have effects on traffic flow and driver behaviors [3,15]. The pedestrian–vehicle interaction under different environment conditions will influence traffic characteristics and various models of signalized intersections, including the capacity model [12], delay model [11], and signal control model [13,14], thus affecting the setting condition of pedestrian phase.

Delay is usually used as a vital performance measure to quantitatively assess pedestrian–vehicle interactions [11]. It is difficult to obtain the real-life crossing delay, thus, theoretical delay estimation methods were applied for signal design and performance evaluation [26]. The early delay model was established by Adams [27], followed by many extensions by Webster [28], Akcelik [29], and the *Highway Capacity Manual* [30]. The *Highway Capacity Manual*, as the most commonly used model, improved the previous model with the assumption of a certain vehicle yielding rate to estimate average control delay [30]. However, these models may not fully reflect the real-life crossing behavior at urban intersections [11]. Other observational studies have found essential factors that should be taken into the delay model, such as traffic flow patterns [31], vehicle yielding rates [32], and pedestrian yield behavior [33]. These factors capture the influence of signal control, phase design, and traffic conditions, which is more consistent with the real-life situation. To better estimate the delay, pedestrian–vehicle interaction should be further quantified, particularly in city CBD areas with a large pedestrian flow. Li et al. [34] proposed a delay model in developing cities, which considers the pedestrian noncompliance and green phase conflict delay. Marisamynathan et al. [35] established pedestrian delay models, which consider the mixed traffic flow, pedestrian arrival rate, and noncompliance rate.

### 2.2. Transportation Equity Research

Transportation equity, in concept, refers to the equal access of multimode participants to economic opportunity by providing equitable levels of accessibility from both horizontal and vertical perspectives [36]. These definitions have provided a foundation upon which to develop approaches for quantifying potential and existing equity impacts of transportation planning, investments, and systems [37]. On the basis of these definitions, studies have been conducted to evaluate the transportation systems on traffic management, cost analysis, accessibility, environmental impact, safety, etc. [38,39,40,41]. Some efforts considering equity have focused on the experiences of transportation planners and their planning practices [42], and others have integrated transportation equity into regular approaches. Vossen et al. [43] focused on an equity compensation mechanism in traffic flow management for various participants. Ferguson et al. [44] integrated equity considerations into the transit frequency-setting problem. Feng et al. [45] proposed an integrated multiobjective model to evaluate the tradeoff between mobility and equity maximization considering environmental capacity. Chen et al. [46] developed a new methodology for rural transportation management considering the equity and cost factors under multiple objectives.

Pedestrians and vehicles, as two main traffic participants in urban intersections, are often involved in the competition for time–space resources. Transportation improvements inevitably lead to an uneven distribution of participant benefits [40]. In other words, assigning excessive green time to vehicles may lead to an increase in pedestrian delay. It indicates the importance of improving pedestrian–vehicle equity at intersections. Pedestrians and vehicles need comparatively fair time–space resources when crossing the intersection. However, limited studies have been conducted to develop quantitative standards to determine the proper pedestrian phase for ensuring transportation equity between pedestrians and vehicles [7].

There are multiple transportation equity approaches developed in academic research, although a gap exists between knowing these approaches and integrating them into real-life practice [37,40]. In contrast to the rich approaches and definitions of traffic equity, engineers have known little about how these methods can be utilized with the constraints of the existing transportation conditions. In practice, the pedestrian phase patterns are often determined by the experience of traffic engineers. One bottleneck appears to be the selection of proper solutions to ensure pedestrian crossing capacity and maintain an acceptable service level for vehicles. Specifically, in urban core business districts with large pedestrian volumes, the study of the *EPP* setting conditions through modeling traffic equity is urgent. Therefore, based on transportation equity and cost analysis, this study proposed a *TEI* and established a bi-objective optimization framework to determine pedestrian phase patterns. Cycle length and green times were optimized in two environment conditions under the *TWC* and the *EPP*.

## 3. Model Formulations

**Figure 1:**
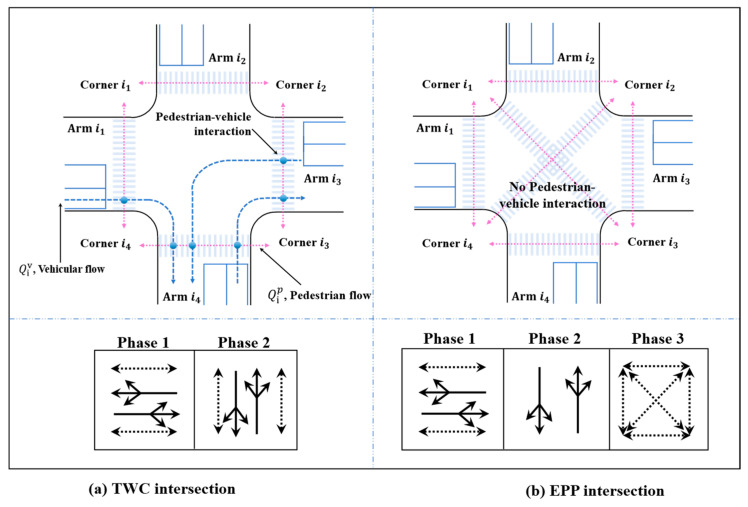
Intersection layout of under *TWC* and *EPP* phase patterns.

The *EPP* gives pedestrians exclusive access to cross the intersection by eliminating the conflicts between pedestrians and vehicles, which can ensure pedestrian safety during the crossing process. However, on the efficiency side, the *EPP* may contribute to higher pedestrian and vehicular delay compared with the *TWC* (see Figure 1). Therefore, the selection of *EPP* and *TWC* is essential to the overall intersection performance as well as individual mobility experience. In this study, a bi-objective optimization model was developed based on transportation equity and monetary cost to obtain the optimal signal timing under wet and dry environment conditions. Based on this, the setting condition of the *EPP* was determined.

### 3.1. Objective Functions

Pedestrians and vehicles need comparatively fair time–space resources when crossing the intersection. In the construction of the transportation equity model, this study proposed the *TEI* to quantify the individual differences under various environment conditions by converting vehicles into passengers per vehicle. More specifically, *TEI* measures the difference in flow rate between pedestrians and vehicles while considering the physical environment in the crossing process. In the overall optimization framework, the primary objective is to minimize the *TEI* to ensure that pedestrians and vehicles can cross the intersection with comparatively fair time–space resources of road per capita. The secondary objective is to minimize the total monetary cost of traffic efficiency and safety. Both traffic safety and efficiency factors are converted into monetary values to select the pedestrian phase pattern in the economic framework [9]. The primary and secondary objective functions were formulated as follows:

(1) $$min\left(TEI\right)=min{\left(\frac{\sum^{\;}q_{i}^{v}}{e^{v}\sum^{\;}T_{v}}-\frac{\sum^{\;}q_{i}^{p}}{e^{p}\sum^{\;}T_{p}}\right)}^{2},$$

(2) $$min\left(M_{D}+M_{S}\right),$$

where $e^{v},\;e^{p}$ is the environmental variable of vehicles and pedestrians, obtained from the field data; $q_{i}^{v}$ is the vehicle flow on arm *i* in the current cycle, (pcu/h) (see Figure 1); $q_{i}^{p}$ is the pedestrian flow on arm *i* in the current cycle, (ped/h); $T_{v}$ is vehicle throughput in this cycle; $T_{p}$ is pedestrian throughput in this cycle; $M_{D}$ is the total monetary delay cost caused by vehicular delay and pedestrian delay; and $M_{S}$ is the total monetary safety cost, due to normal exposures and pedestrian noncompliance ratio. The key notations used in this paper are summarized in Table A1, Appendix B.

### 3.2. Signal Constrains

The minimal and maximal values of the cycle length ($C_{min}$ and $C_{max}$), were determined according to the *Traffic Signal Timing Manual*. The cycle length was constrained by:

(3) $$C_{min}\leq C\;\leq C_{max},$$

The green split should satisfy the minimal green time requirement for pedestrians and vehicles in each phase. The green split is constrained by:

(4) $$max\left\{g_{i,min}^{p}\right\}\;\leq g_{x},\forall x,$$

(5) $$max\left\{g_{i,min}^{v}\right\}\;\leq g_{x},\;\forall x,$$

The sum of green times and intergreens ($I_{x}$) in all phases were required to meet the cycle length and were constrained by:

(6) $$C\;\leq g_{x},$$

(7) $$C=\underset{x}{\sum }(I_{x}+g_{x}),$$

### 3.3. Transportation Equity Modeling

The primary objective was to ensure fair crossing capacity between vehicles and pedestrians in pedestrian phase patterns. In this study, *TEI* was assessed by the difference in the flow ratio between vehicles and pedestrians, in which the flow ratio was modified by the environment coefficient. Based on the previous study on the crosswalk traffic capacity [47,48], vehicle and pedestrian throughput per cycle under the *TWC* and *EPP* conditions were further adjusted in this study. To obtain a more comprehensive capacity of signalized intersection, this study carried out capacity modification under the *TWC* and *EPP* conditions.

#### 3.3.1. Pedestrian Throughput

Under the *TWC* control, the right of way was simultaneously allocated to vehicles and pedestrians at the same phase. The interaction between pedestrians and right-turn vehicles could contribute to the degraded throughput and potential traffic conflicts. Under the *TWC* condition, pedestrian throughput could be affected by right-turn vehicles brought by the exclusive right-turn lane. Based on the traditional crosswalk capacity model [48], this study considered the influence of right-turn vehicles on the pedestrian crossing process. To be consistent with the actual situation, the proposed pedestrian throughput model introduced the reduction coefficient of the exclusive right-turn lane on pedestrians $\left(K_{p}\right)$. Henceforth, pedestrian crossing throughput was calculated as follows:

(8) $$T_{p}=K_{p}\left(\frac{t_{p}-\frac{l}{v_{p}}-t_{2}}{\frac{b_{1}}{v_{p}}}+1\right),$$

where $t_{P}$ is the green time of pedestrian signal; l is the pedestrian crosswalk length; $b_{1}$ is the distance between pedestrians; $v_{p}$ is the speed of crossing pedestrians; $t_{2}$ is the lost time due to pedestrian safety concerns at the end of the red light; $K_{p}$ is the reduction coefficient of the exclusive right-turn lane on pedestrians under *TWC*, $K_{p}=1-Q_{R}t_{R}/C$; C is the cycle length; $t_{R}$ is the time when the right-turning vehicle occupies the sidewalk, $t_{R}=(l_{C}+l_{R)}/v_{R}$; $l_{C}$ is the converted vehicle length; $l_{R}$ is the minimum safe distance between pedestrians and right-turn vehicles; and $v_{R}$ is the speed of right-turn vehicles.

Under the *EPP* control, pedestrian crossing throughput ($T_{p}$) was divided into two parts by period: the *EPP* period $\left(t_{E}\right)$ and the non-*EPP* period $\left(C-t_{E}\right)$. During the *EPP* period $\left(t_{E}\right)$, $T_{p1}$ was the corresponding pedestrian crossing throughput from all directions (including diagonal lines) in each cycle. During the non-*EPP* period $\left(C-t_{E}\right)$, pedestrians could still cross the street as in the *TWC*. Henceforth, $T_{p2}$ was the pedestrian crossing throughput in each cycle during the non-*EPP* period $\left(C-t_{E}\right)$. The total pedestrian crossing throughput under the *EPP* was formulated as follows. The detailed calculations of $T_{p1}$ and $T_{p2}$ are listed in Appendix A.

(9) $$T_{p}=T_{p1}+T_{p2}.$$

#### 3.3.2. Vehicle Throughput

Based on the Design Regulation of Urban Road Engineering (CJJ37—90) in China [49], this study established the vehicle throughput model using the basic idea of the stop line method under both the *TWC* and *EPP* conditions. The stop line method is one of the commonly used methods to calculate the traffic capacity of signalized intersections in China.

Under the *TWC* control, the total vehicle throughput consisted of three parts: throughput from left turn, straight, and right-turn lanes. The total vehicle crossing throughput was calculated as follows:

(10) $$T_{v}=\frac{t_{\mathrm{gL}}-\frac{v_{s}}{2a_{s}}}{K_{1}t_{L}}+K_{p}\frac{t_{\mathrm{gT}}-\frac{v_{s}}{2a_{s}}}{t_{T}}+\frac{C}{K_{1}t_{R}\left(1-K_{2}\right)},$$

where $t_{\mathrm{gL}},\;t_{\mathrm{gT}}$ are the signal period of left turn and go straight in a cycle, respectively; $t_{L},\;t_{T},\;t_{R}$ are the average vehicle headway of left turn, straight, and right turn, respectively; $K_{1},\;K_{1}^{\prime }$ are the adjustment coefficients of the turning vehicle headway under *TWC* and *EPP*; $K_{2},\;K_{2}^{\prime }$ are the reduction coefficients of the vehicle volume decrease caused by the conflict between pedestrians and right-turn vehicles under the *TWC* and *EPP*; $v_{s}$ is the speed of vehicles; and $a_{s}$ is the acceleration of vehicles.

Under the *EPP* control, vehicles were not allowed to cross during the *EPP* period in a cycle. The vehicle throughput in the rest of the cycle $\left(C-t_{E}\right)$ was as follows:

(11) $$T_{v}=\frac{t_{\mathrm{gL}}\frac{\left(C-t_{E}\right)}{C}-\frac{v_{s}}{2a_{s}}}{K_{1}^{\prime }t_{L}}+K_{p}^{\prime }\frac{t_{\mathrm{gT}}\frac{\left(C-t_{E}\right)}{C}-\frac{v_{s}}{2a_{s}}}{t_{T}}+\frac{C-t_{E}}{K_{1}^{\prime }t_{R}\left(1-K_{2}^{\prime }\right)}.$$

### 3.4. Cost Modeling

Traffic factors can be converted into monetary values as economic criteria to select the pedestrian phase pattern. Traffic efficiency factors were measured with a new delay model considering vehicle yielding rate under the *TWC* and the *EPP*. Traffic safety factors were assessed by modeling the pedestrian–vehicle exposures and pedestrian violations. To calculate the monetary cost of pedestrian and vehicle delay, we estimated the average pedestrian/vehicle delay per hour using the UAV-based traffic datasets and applied the unit average cost of pedestrian/vehicle delay ($C_{p}/C_{v})$ to estimate the equivalent monetary value of wasted pedestrian/vehicle time.

The total delay cost of traffic participants consisted of two parts, the vehicle delay cost and the pedestrian delay cost. Pedestrian–vehicle interaction existed under the *TWC* condition, therefore, the vehicle/pedestrian delay $(d_{\mathrm{PVI}}^{v}/d_{\mathrm{PVI}}^{p}$) during pedestrian–vehicle interaction were considered in the monetary evaluation. The total delay cost under *TWC* $\left(M_{D}^{TWC}\right)$ was calculated with the following equation:

(12) $$M_{D}^{TWC}=C_{p}\underset{i}{\sum }\left[Q_{i}^{p}\cdot \alpha_{ij}\cdot (d_{\mathrm{ij}}^{p}+d_{\mathrm{PVI}}^{p})\right]+C_{v}\cdot N_{vp}\underset{i}{\sum }\left(d_{i}^{v}\cdot Q_{i}^{v}+d_{\mathrm{PVI}}^{v}\right),$$

where $C_{p}$ is the unit average delay cost of one pedestrian per hour ($/h); $C_{v}$ the unit average delay cost of one vehicle per hour ($/h); $N_{vp}$ is the average number of passengers carried by vehicles, (ped/pcu); $Q_{i}^{v}$ is the total vehicular demand at corner *i*, (pcu/h); and $Q_{i}^{p}$ is the total pedestrian crossing demand at corner *i*, (ped/h).

Under the *EPP* condition, there was no pedestrian–vehicle interaction at the intersection. Only control delay caused by traffic signals was included in the pedestrian delay. Meanwhile, vehicle delay included the control delay and delay caused by *EPP*. $g_{E}$ is the green time of the *EPP* (s). The total delay cost under the *EPP* ($M_{D}^{EPP}$) was calculated as follows:

(13) $$M_{D}^{EPP}=C_{p}\underset{i}{\sum }\left(Q_{i}^{p}\cdot d_{\mathrm{ij}}^{Sp}\right)+C_{v}\cdot N_{vp}\underset{i}{\sum }\left[\left(g_{E}+d_{i}^{v}\right)\cdot Q_{i}^{v}\right].$$

The total safety cost $\left(M_{S}\right)$ consists of safety costs due to accidents $\left(M_{S}^{I}\right)$ and pedestrian compliance ratio $\left(M_{S}^{II}\right)$. It can be calculated with the following equation:

(14) $$M_{S}=M_{S}^{I}+M_{S}^{II}.$$

#### 3.4.1. Pedestrian–Vehicle Interaction

This study adopted the delay estimation model of *Highway Capacity Manual* (2010) to estimate the vehicle control delay at signalized intersections [50]. Pedestrian delay under the *TWC* control consisted of signal delay, conflict delay, and detour delay. The detailed algorithm for the delay model is listed in Appendix A.

Vehicle delay during the pedestrian–vehicle interaction $\left(d_{\mathrm{PVI}\;}^{v}\right)$ can be caused by the deceleration and the interaction process, which was calculated as:

(15) $$d_{\mathrm{PVI}\;}^{v}=\left(t_{pv}^{v}+\frac{v_{0}-\sqrt{v_{0}^{2}+2a_{c}L}}{a_{c}}-\frac{L}{V_{0}}\right)\cdot N\cdot y_{v},$$

where $t_{pv}^{v}$ is the pedestrian–vehicle interaction time of vehicle, obtained from field study; $v_{0}$ is the initial vehicle velocity when interaction occurs, (m/s); L is the distance between vehicle and pedestrian when pedestrian–vehicle interaction occurs, (m); $a_{c}$ is the acceleration when the vehicle decelerates, $\left(m/s^{2}\right)$; and $y_{v}$ is the vehicle yielding rate.

Pedestrian delay during the pedestrian–vehicle interaction $\left(d_{\mathrm{PVI}}^{p}\right)$ can be caused by the interaction time and condition, which was calculated as:

(16) $$d_{\mathrm{PVI}}^{p}=\left(t_{pv}^{p}-\frac{d}{v_{p}}\right)N\cdot y_{p},$$

where d is the crosswalk length; $t_{pv}^{p}$ is the pedestrian–vehicle interaction time of pedestrian; $y_{p}$ is the pedestrian yielding rate; and N is the number of pedestrian–vehicle interactions before setting the *EPP* control.

#### 3.4.2. Safety Cost

Considering the pedestrian crossing danger, traffic exposures and the pedestrian noncompliance ratio are two primary factors that affect intersection safety [3]. Therefore, the total safety cost $\left(M_{S}\right)$ consists of the safety cost due to accidents $\left(M_{S}^{I}\right)$ and the pedestrian compliance ratio $\left(M_{S}^{II}\right)$. In the potential accidents analysis based on exposures, this study adopted the exposure calculation method from Nilsson [51], which has been applied in many related studies [9,52]. Based on this method, this study divided the calculation into two conditions. Under the *EPP* condition, the intersection exposure was zero because no vehicle interaction existed during the pedestrian crossing process. Under the *TWC* condition, the intersection exposure depended on the volume of pedestrians and turning vehicles that may become involved in a conflict with the pedestrians. In safety cost caused by traffic accidents, the accident probability distribution was used to assess the traffic safety under various pedestrian phase patterns [53]. The monetary value of safety costs due to accidents $\left(M_{S}^{I}\right)$ can be calculated as follows:

(17) $$M_{S}^{I}=\delta \cdot C_{A}\overset{4}{\underset{i=1}{\sum }}B_{i},$$

where $B_{i}$ is the number of potential traffic accidents under the *TWC* pattern and $C_{A}$ is the average cost of an accident ($/accident).

Most pedestrian violations are caused by noncompliance behavior of pedestrians, such as crossing through a red light. To reflect the real-life condition, this study introduced the transformation coefficient ($P_{T}^{2}$) from the noncompliance ratio to accidents based on a safety evaluation method from Yuan [54]. The monetary value of safety cost due to pedestrian violations $\left(M_{S}^{II}\right)$ can be calculated with the following equation:

(18) $$M_{S}^{II}=C_{A}\cdot P_{T}^{2}\cdot \overset{4}{\underset{i=1}{\sum }}Q_{i}^{p}\cdot \alpha_{ij}\cdot \rho ,$$

where $P_{T}^{2}$ is the average accident number to pedestrian noncompliance ratio; ρ is the estimated probability of pedestrian noncompliance; $Q_{i}^{p}$ is the total pedestrian crossing demand at corner *i*; and $\alpha_{ij}$ is the proportion of pedestrian volume from corner *i* to another in total pedestrian demand of corner *i*.

## 4. Preliminaries: Data and Methods

### 4.1. Solution Algorithms

The proposed optimization model was formulated as a mixed-integer nonlinear programming (MINLP) model with a bi-objective structure. The model has nonlinear, nonconvexity, and nondifferential characteristics, which make it difficult to find the global optimal solution through traditional nonlinear programming methods. The nondominated sorting genetic algorithm II (NSGA-II) is a commonly used algorithm in solving MINLP problems [13,55,56]. This study applied the NSGA-II for yielding optimal cycle length and green time. The performance of the NSGA-II was compared with that of the multiobjective evolutionary algorithm based on decomposition (MOEA). The MOEA explicitly decomposed the multiobjective problems into scalar optimization subproblems and has been demonstrated to be effective for solving two- and three-objective problems [57]. The abbreviations in this study are listed in Abbreviations section.

### 4.2. Data Resource

**Figure 2:**
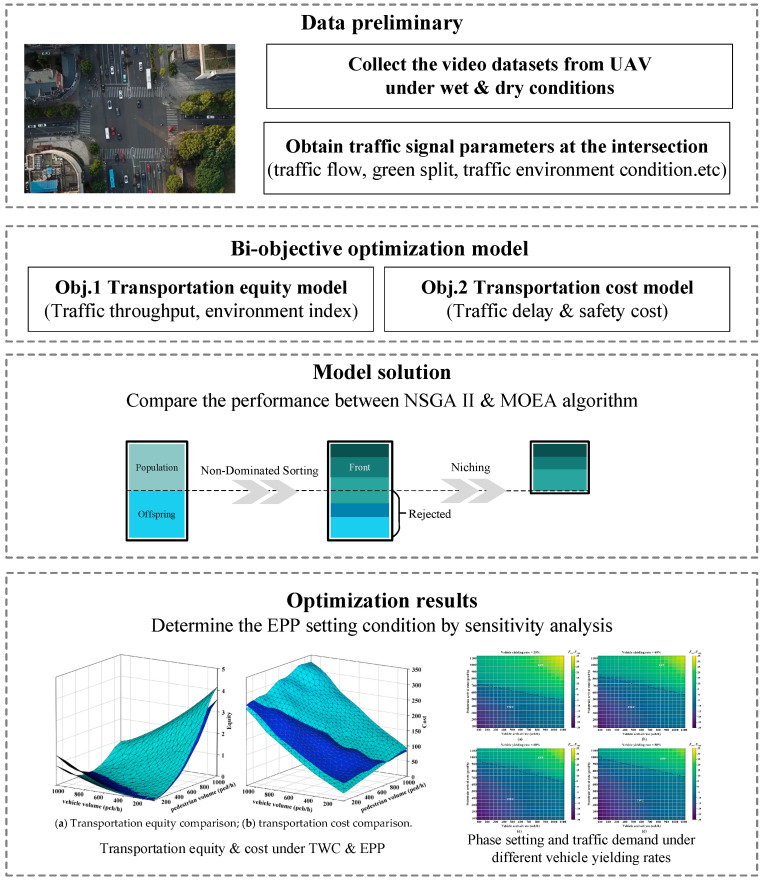
The proposed optimization framework.

An observational study was conducted to investigate the *EPP* setting condition at a signalized intersection (Huangxin Rd. and Guoquan Rd.) located in Shanghai, China. The dataset contained in this research came from videos acquired from an unmanned aerial vehicle (UAV). Considering the difference in environment perception between pedestrians and drivers, UAV-based datasets were collected under two conditions. The first condition was set at the morning peak hours of a dry and sunny day, while the second condition is set at the morning peak hours of a wet and rainy day. Traffic parameters were calibrated from the traffic datasets extracted from the UAV-based videos (see Table 1). The proposed model was also implemented with this field data to demonstrate its applicability. The overall framework of this study is illustrated in Figure 2.

## 5. Numerical Examples and Sensitivity Analysis

In this section, experiments were carried out to answer the following research questions: 1. What are the characteristics of the nondominated sets constructed by the proposed signal optimization methods? 2. Why is transportation equity important for signal timing optimization? 3. What is the impact of transportation equity and pedestrian–vehicle interaction on the *EPP* setting?

### 5.1. Metaparameter Analysis

To compare the modeling performance under both the *EPP* and the *TWC* conditions, the initial traffic parameters are summarized in Table 1. Empirical parameters were obtained from previous studies [53,58]. The average cost of each accident ($C_{A}$) was according to China Statistical Yearbook 2019. The unit average cost of pedestrian delay ($C_{p}$) and the unit average cost of vehicle delay ($C_{v}$) were referred to the previous cost-related studies in China [54,59].

As mentioned in the solution algorithms, the performance of NSGA-II is compared with that of MOEA. To make the tuning process computationally, a grid search for optimal parameter values was performed based on a multiobjective optimization framework [60]. The hypervolume indicator (*I_H_*) was calculated to evaluate the optimization performance of NSGA-II and MOEA. The larger the *I_H_*, the better the algorithm performance will be. The best out of optimization runs with 3000 objective evaluations were selected for each configuration. For NSGA-II, the overall best configuration was *P_s_*/*M_r_*/*C_r_* = 50/0.05/0.3, while the configuration *P_s_*/*M_r_*/*C_r_* = 50/0.05/0.3 was the top performer for MOEA.

**Figure 3:**
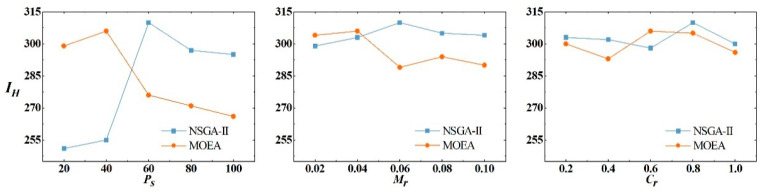
The influence of changing the metaparameters on *I_H_*.

To evaluate the effects of individual metaparameters, a metaparameter analysis was conducted based on the best configuration. For a configuration with a specific value of the observed individual metaparameter, *I_H_* was computed by changing the value of the interest parameter. Figure 3 presents how changes in one parameter affect the optimization performance when fixing the other parameters at their best configurations. It can be concluded that *P_s_* has a stronger influence on *I_H_* than *M_r_* and *C_r_* in the NSGA-II algorithm. NSGA-II does not rely on an external archive; thus, a large population indicates rich initial diversity. In this case, constructing a large enough pool of nondominated solutions was possible. In contrast, MOEA prefers smaller *M_r_* because the neighborhood mechanism in MOEA accelerates the propagation of mutation across the population.

### 5.2. Optimization Results

The final experiments were run on a desktop computer with an Intel i7 CPU and 16 GB of RAM. The analysis was performed with the Pymoo package under the Python 3.8 environment developed by Python Software Foundation in Netherlands. In this section, the nondominated sets constructed by MOEA and NSGA-II are compared.

**Figure 4:**
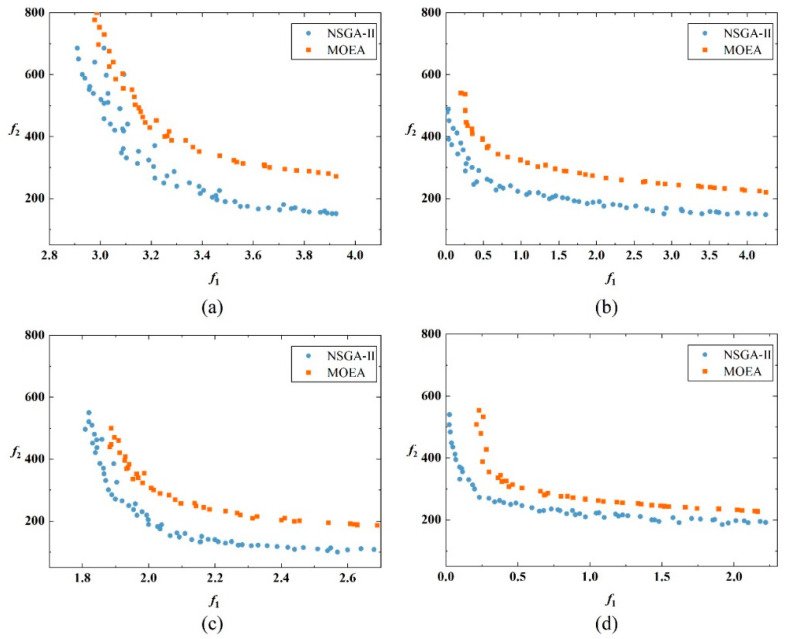
Nondominated fronts constructed by NSGA-II and MOEA for the corresponding conditions: (**a**) *TWC* in wet condition; (**b**) *EPP* in wet condition; (**c**) *TWC* in dry condition; (**d**) *EPP* in dry condition (*f*_1_: equity, *f*_2_: cost).

Figure 4 shows the nondominated fronts for the four conditions. In the experiments, NSGA-II outperformed MOEA both in *I_H_* and in the spread of the nondominated front. The reason for the poor performance of MOEA is the inherited replacement strategy that can result in limited population diversity and premature convergence. In contrast, NSGA-II had better global exploration capabilities than MOEA. According to the distribution of nondominated fronts of the two methods, the fronts are approximately convex, with two objectives changing in reverse. It indicates that signal optimization methods lacking cost consideration may lead to transportation inequity between pedestrians and vehicles. The nondominated fronts obtained by the proposed model cover a spectrum of solutions that balance either of the two objectives.

**Figure 5:**
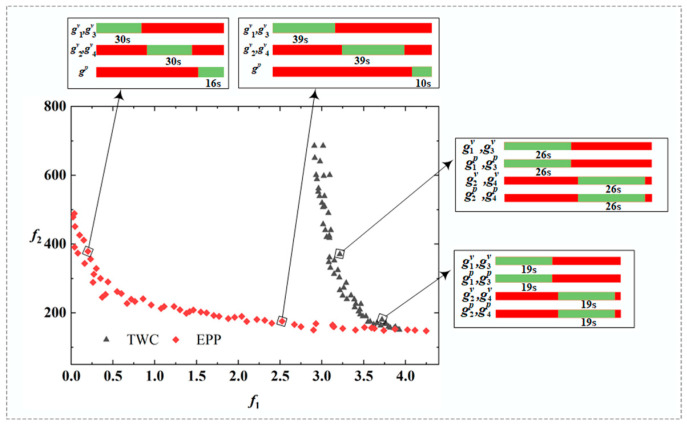
The nondominated front and signal timing pattern under *EPP* and *TWC* conditions.

In terms of environment conditions and the *EPP* setting, conclusions can be drawn from Figure 5. Compared with the *TWC*, one fundamental advantage of *EPP* is that it improves the transportation equity between pedestrians and vehicles by increasing the green time for pedestrians (see Figure 5). In the first condition (wet and rainy), the cost of *TWC* was higher than in the second condition (dry and sunny) (see Figure 4a,c). This is in line with the conclusion that poor weather conditions can lead to degraded road capacity, thus increasing the cost. After setting the *EPP*, the cost of traffic participants decreased under the first condition because the *EPP* can eliminate the interaction between pedestrians and vehicles. However, under the second condition, the cost slightly increased under the *EPP* control (see Figure 4d). One possible explanation is that the traffic demand under the second condition failed to meet the threshold of the *EPP* setting. Such phenomena will be further discussed in the next section using sensitivity simulations.

### 5.3. Sensitivity Analysis

Pedestrian volume, vehicle volume, and yielding rate are three primary parameters influencing the setting conditions of the *EPP*. This section performs a sensitivity analysis of the proposed model with respect to the yielding rate under two environment conditions. The changes in environment conditions influence the decision-making process of pedestrians and vehicles [24]. Considering the pedestrian–vehicle interaction, the sensitivity analysis was carried out, with the vehicle yielding rates of 0.2, 0.4, 0.6, and 0.8.

**Figure 6:**
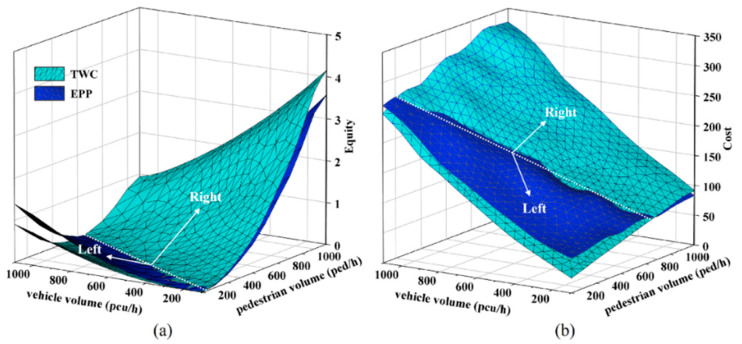
Impacts of pedestrian and vehicle demand: (**a**) Transportation equity comparison; (**b**) transportation cost comparison.

Figure 6 illustrates the impact of pedestrian and vehicle volumes on the *EPP* and the *TWC* performances with respect to transportation equity and cost under the first condition. Figure 6a shows the change of setting conditions under varying pedestrian and vehicle volumes in terms of transportation equity. On the right side of the white dotted line, the values of *TEI* under the *EPP* are lower than that of the *TWC*, while the values of *TEI* under the *EPP* are higher on the left side. In other words, a lower value of *TEI* suggests less pedestrian–vehicle difference, indicating fairer interaction. This indicates that under high traffic volumes, the *EPP* can provide better transportation equity than the *TWC*. Figure 6b reveals the change of setting conditions under different pedestrian and vehicle volumes regarding transportation cost. On the right side of the white dotted line, the cost of the *EPP* is lower than that of the *TWC*, while the cost of the *EPP* is higher on the left side. In low traffic demand conditions, the cost of the *EPP* exceeded that of the *TWC*, while, with high traffic demand, the cost of *TWC* exceeded that of the *EPP*. When the pedestrian and vehicle volumes increased, the cost increased under both the *EPP* and *TWC* conditions. In other words, under high traffic volumes with high pedestrian volumes, the setting of the *EPP* was more cost beneficial than the *TWC*.

To analyze the combined impact of the two objectives (transportation cost and equity) on the *EPP* setting, the bi-objective model was transformed into a single objective model. The detailed transformation formulation is based on the pseudoweight vector approach listed in Appendix A.

**Figure 7:**
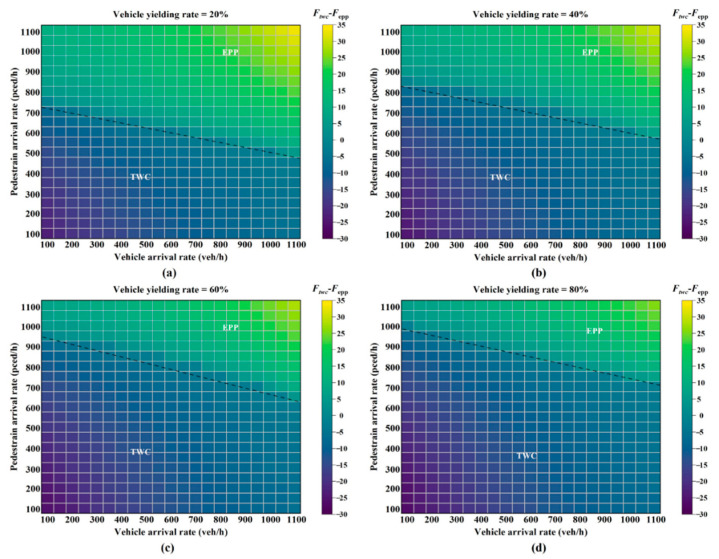
Phase setting and traffic demand under different vehicle yielding rates in the first condition: (**a**) vehicle yielding rate, 20%; (**b**) vehicle yielding rate, 40%; (**c**) vehicle yielding rate, 60%; (**d**) vehicle yielding rate, 80%.

**Figure 8:**
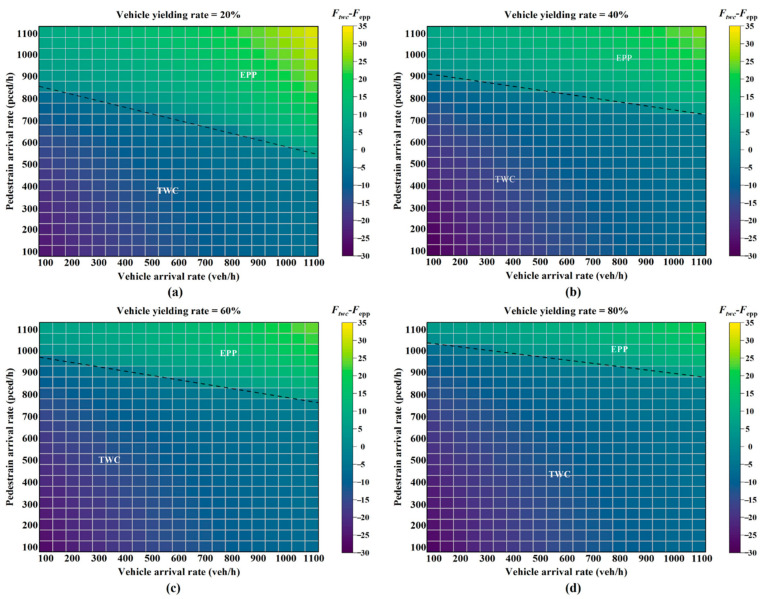
Phase setting and traffic demand under different vehicle yielding rates in the second condition: (**a**) vehicle yielding rate, 20%; (**b**) vehicle yielding rate, 40%; (**c**) vehicle yielding rate, 60%; (**d**) vehicle yielding rate, 80%.

Figure 7 and Figure 8 indicate the *EPP* setting domain with respect to traffic volume and yielding rate in two different environment conditions. The black dotted line (the zero-difference line) divides the phase setting domain into two parts: the *TWC* and the *EPP* application. When the line takes on a negative difference, the *TWC* is better than the *EPP*, while the *EPP* is better when the difference is positive.

When the yielding rate increased, the boundary condition for setting the *EPP* became loose under both environment conditions, as shown in Figure 7 and Figure 8. When the yielding rate was 0.2 (see Figure 7a), the *EPP* was more suitable for a pedestrian demand higher than 750 ped/h and a vehicle demand higher than 800 pcu/h. When the yielding rate was 0.8, as shown in Figure 7d, few pedestrian–vehicle interactions emerged. In this case, the *EPP* setting was recommended when the pedestrian demand exceeded 1000 ped/h and the vehicle demand exceeded 900 pcu/h.

With the same yielding rate, the *EPP* setting criteria in the second condition was stricter than the criteria in the first condition, as compared with Figure 7 and Figure 8. In other words, the *EPP* is more suitable than the *TWC* at intersections with poor weather conditions for preventing potential pedestrian–vehicle conflicts. In Figure 7a and Figure 8a, with the yielding rate of 0.2, the *EPP* is suitable when pedestrian demand reaches 750 ped/h in the first condition. However, 880 ped/h is required in the second condition. Based on the results of the sensitivity analysis, this study concludes:

- When the vehicular volume is constant, the *EPP* setting has a better effect with high pedestrian volumes.
- With the same traffic demand, it is more suitable to set the *EPP* when the yielding rate is low. The analysis shows that the yielding rate has a significant impact on the *EPP* setting conditions at intersections.
- Environment factors have effects on the *EPP* setting condition. With the same traffic demand and yielding rate, the *EPP* is more suitable under wet conditions. It shows that incorporating the environment condition into the *EPP* setting criteria is essential.

## 6. Conclusions

This study established a bi-objective optimization model to determine the *EPP* setting criteria, with consideration for the tradeoff between transportation equity and cost. The equity model was established to minimize the individual differences under different environment conditions. The cost model considers the pedestrian–vehicle interaction and converts traffic safety and efficiency factors into monetary values, which minimize the delay and safety costs. A nonlinear programming model was formulated, and the optimal signal timing was obtained by solving the proposed model. To validate the optimization framework, this study conducted a numerical experiment based on the UAV datasets collected under two environment conditions. The algorithm performance of two evolutionary algorithms (i.e., the NSGA-II and the MOEA) were compared.

The results of the numerical experiment showed that transportation equity was inversely related to the cost, indicating that the decrease in transportation equity leads to an increased cost. The obtained Pareto front approximations corresponded to diverse pruning solutions and balance between optimizing either objective to different extents. The numerical analysis and field study validated the effectiveness of the proposed model in improving the comprehensive performance of the intersections. The results revealed the tradeoff between transportation equity and cost, indicating the necessity to optimize the pedestrian phase selection strategy, rather than having a one-sided consideration.

Considering the influence of vehicular/pedestrian demand, yielding rate, and environment conditions on the application domains of the *EPP*, the sensitivity analysis was conducted based on the numerical experiment. The results showed that the *EPP* was more suitable at intersections with a high pedestrian volume, especially in the first environment condition. Additionally, the application domains of the *EPP* change significantly with the yielding rate. Thus, it is essential to incorporate the yielding rate and the environment variable into the *EPP* setting criteria.

The research results provide operation guidance for decision-makers to better select pedestrian phase patterns at signalized intersections. In addition to the pedestrian–vehicle interaction and environment factors, traffic equity modeling can be further optimized by considering multimodal transportation and the accessibility of traffic participants. Extensive field studies should be conducted to further calibrate the cost values and environment variables under various traffic demand patterns and intersection configurations. Another possible extension is to study the pedestrian phase setting, with consideration for the linkage control of adjacent signalized intersections.

## Figures and Tables

**Table 1:** Parameters in model validation.

Notation	Definition	Value
**Monetary Parameters**		
$C_{v}$	Unit average delay cost of one vehicle per hour ($/h)	6
$C_{p}$	Unit average delay cost of one pedestrian per hour ($/h)	4
$C_{A}$	The average cost of an accident ($/accident)	65,000
$P_{T}^{1}$, $P_{T}^{2}$	Average accident number to pedestrian noncompliance ratio	0.00286
$K_{p}^{\prime }$	Reduction coefficient of the exclusive right-turn lane on pedestrians under *TWC*	0.6
ρ	Probability of pedestrian noncompliance	0.25
**Crossing Parameters**		
l	Pedestrian crosswalk length, (m)	15
$l_{3}$	Diagonal crosswalk length, (m)	28
d	Crosswalk width, (m)	5
$b_{1}$	Distance between pedestrians, (m)	0.75
$l_{C}$	Converted vehicle length, (m)	6
$l_{R}$	Minimum safe distance between pedestrians and right-turn vehicles, (m)	0.8
**Signal Parameters**		
$g_{i,min}^{v}$	Minimal green time for vehicles (s)	10
$C_{min}$	Minimum cycle length, (s)	34
$C_{maz}$	Maximum cycle length, (s)	200
$t_{E}$	*EPP* period, (s)	26
$t_{2}$	Lost time due to pedestrian safety concerns at the end of the red light, (s)	2
t	Minimum length of the acceptable gap for crossing (s)	5
**Vehicle & Pedestrian Parameters**		
$v_{p}$	Speed of crossing pedestrians, (m/s)	1.2
$v_{R}$	Speed of right-turn vehicles, (m/s)	2.78
$\mu_{i}$	Average flow rate of turning vehicles I, (pcu/s)	0.14
$q_{i}^{p}$	Pedestrian flow on arm *i* in the current cycle, (ped/h)	2000
$q_{i}^{v}$	Vehicle flow on arm *i* in the current cycle, (pcu/h)	1000

## Data Availability

The dataset presented in this article is not readily available because it is a part of the ongoing projects (The Science and Technology Commission of Shanghai Municipality); therefore, the dataset is confidential during this period.

## Abbreviations

*EPP*	Exclusive Pedestrian Phase
*TWC*	Traditional Two-way Control Phase
*TEI*	Transportation Equity Index
MOEA	Multiobjective Evolutionary Algorithm based on Decomposition
NSGA-II	Nondominated Sorting Genetic Algorithm II
MINLP	Mixed Integer Nonlinear Programming
UAV	Unmanned Aerial Vehicle

## Appendix A

In Section 3.3.1, under the *EPP* control, pedestrian crossing throughput ($T_{p}$) was divided into two parts by period. During the *EPP* period $\left(t_{E}\right)$, $T_{p1}$ is the corresponding pedestrian crossing throughput from all directions (including diagonal lines) in each cycle. $T_{p2}$ is the pedestrian crossing throughput in each cycle during the non-*EPP* period $\left(C-t_{E}\right)$.

(A1) $$T_{p1}=2\cdot \left(N_{1}+N_{2}+N_{3}\right),$$

(A2) $$T_{p2}=K_{p}^{\prime }\frac{t_{p}\frac{C-t_{E}}{C}-\frac{l}{v_{p}}-t_{2}}{\frac{b_{1}}{v_{p}}},$$

$N_{1},\;N_{2},\;N_{3}$ is the number of pedestrians passing through the crosswalks in each cycle, with the crosswalk length of $l_{1},\;\;l_{2}$, $l_{3}$, $N_{i}=n(t_{E}v_{p}-l_{i})/b_{1}$; $t_{E}$ is the *EPP* period with the measured value of 26 s in the case study; $t_{P}$ is the length of green for pedestrians to cross the street; $t_{s}$ is the minimum time for pedestrians to cross the street, $t_{s}=l_{i}/v_{p}$; $l_{1},\;l_{2}$ is the crosswalk length; $l_{3}$ is the diagonal crosswalk length; n is the number of pedestrians in the first row at the initial stage of *EPP*, n=d/0.75; d is the crosswalk width; $K_{p}^{\prime }$ is the reduction coefficient of straight vehicles affected by left-turn vehicles under the *EPP* control.

In Section 3.4.1, the vehicle delay consisted of uniform delay and incremental delay and is given as follows:

(A3) $$d_{i}^{v}=\frac{0.5C{\left(1-\frac{g}{C}\right)}^{2}}{1-\left[\min\left(1,X\right)\frac{g}{C}\right]}+900T\left[(X_{A}-1)+\sqrt{{\left(X_{A}-1\right)}^{2}+\frac{8kIX}{C_{A}T}}\right].$$

The pedestrian control delay ($d_{\mathrm{ij}}^{p}$) is given as follows:

(A4) $$d_{\mathrm{ij}}^{p}=\frac{{\left(C-g_{i}^{p}\right)}^{2}}{2C}+\delta \left(\frac{e^{\mu_{i}t}-\mu_{i}t-1}{\mu_{i}}+\frac{l_{1}+l_{2}-l_{3}}{v^{p}}\right),$$

where $(C-g_{i}^{p})$ is pedestrian STOP intervals; $\mu_{i}$ denotes the average flow rate of turning vehicles *i* (veh/s); t is the minimum length of the acceptable gap for crossing (s); δ is a binary variable representing the pedestrian phase pattern, $\delta =\{\begin{array}{c} 0,EPP\\ 1,TWC \end{array}$. Noted that only signal delay exists under the *EPP* condition.

In Section 5.3, the two objectives in the proposed model were weighted by the pseudoweight vector approach proposed in [61] to choose a solution out of a solution set in the context of multiobjective optimization. The pseudoweight $\left(w_{i}\right)$ for the two corresponding objective functions (*f*_1_: equity, *f*_2_: cost) can be calculated by:

(A5) $$w_{i}=\frac{\left(f_{i}^{max}-f_{i}\left(x\right)\right)/\left(f_{i}^{max}-f_{i}^{min}\right)}{\sum_{m=1}^{M}\left(f_{m}^{max}-f_{m}\left(x\right)\right)/\left(f_{m}^{max}-f_{m}^{min}\right)}\;i=1,\;2$$

This equation calculates the normalized distance to the worst solution regarding each objective. Based on this, the bi-objective model was transformed into a single objective model.

## Appendix B

**Table A1:** Notation and parameter description.

Parameter	Description
$e^{v},\;e^{p}$	Environmental index of vehicles and pedestrians
$q_{i}^{v}$	Vehicular flow on arm *i* in current cycle, (pcu/h)
$q_{i}^{p}$	Pedestrian flow on arm *i* in current cycle, (ped/h)
$T_{v}$	Vehicular throughput in this cycle, (pcu/h)
$T_{p}$	Pedestrian throughput in this cycle, (ped/h)
$t_{P}$	Length of green of pedestrian signal, (s)
$l_{1},l_{2}$	Pedestrian crosswalk length, (m)
$l_{3}$	Diagonal crosswalk length, (m)
d	Crosswalk width, (m)
$b_{1}$	Distance between pedestrians, (m)
$v_{p}$	Speed of crossing pedestrians, (m/s)
$t_{2}$	Lost time due to pedestrian safety concerns at the end of the red light, (s)
C	Cycle length, (s)
$g_{E}$	Green time of *EPP*, (s)
$t_{R}$	Time when the right-turning vehicle occupies the sidewalk, (s)
$l_{C}$	Converted vehicle length, (m)
$l_{R}$	Minimum safe distance between pedestrians and right-turning vehicles, (m)
$v_{R}$	Speed of right-turning vehicles, (m/s)
$t_{E}$	*EPP* period, (s)
$t_{P}$	Length of green for pedestrians to cross the street, (s)
$t_{s}$	Minimum time for pedestrians to cross the street, (s)
$t_{pv}^{p}$, $t_{pv}^{v}$	Pedestrian–vehicle interaction time of pedestrian and vehicle, (s)
$N_{1},N_{2},N_{3}$	Number of pedestrians passing through the crosswalks in each cycle with the crosswalk length of $l_{1},\;\;l_{2}$, $l_{3}$;
n	Number of pedestrians in the first row at the initial stage of *EPP*
$t_{\mathrm{gL}},t_{\mathrm{gT}}$	Signal period of left turn and straight in a cycle, respectively
$t_{L},t_{T,}t_{R}$	Average vehicle headway of left turn, straight, and right turn, respectively
$y_{v}$	Vehicle yielding rate (%)
$y_{p}$	Pedestrian yielding rate (%)
N	Numbers of pedestrian–vehicle interactions before setting *EPP*
$v_{s}$	Speed of vehicles, (m/s)
$a_{s}$	Acceleration of vehicles, (m/s^2^)
$Q_{i}^{v}$	Total vehicular demand at corner *i*, (pcu/h)
$Q_{i}^{p}$	Total pedestrian crossing demand at corner *i*, (ped/h)
$B_{i}$	the number of potential traffic accidents under *TWC* control
δ	Binary variable representing the pedestrian phase type, $\delta =\{\begin{array}{c} 0,EPP\\ 1,TWC \end{array}$
$P_{T}^{2}$	Average accident number to pedestrian noncompliance ratio
ρ	Probability of pedestrian noncompliance
$\alpha_{ij}$	Proportion of pedestrian volume from corner *i* to corner *j* in total pedestrian demand of corner *i*
